# Molecular and Morphological Identification and Pathogenicity of *Fusarium* Species Causing Melon Wilt in Russia

**DOI:** 10.3390/jof11120888

**Published:** 2025-12-17

**Authors:** Irina Engalycheva, Elena Kozar, Alina Kameneva, Maria Sletova, Svetlana Vetrova, Vera Chizhik, Maria Kornilova, Viktor Martynov

**Affiliations:** 1 Federal State Budgetary Scientific Institution “Federal Scientific Vegetable Center” (FSBSI FSVC), 143072 Moscow, Russia; kozar_eg@mail.ru (E.K.); alina.malina1290@gmail.com (A.K.); gvina@yandex.ru (M.S.); lana-k2201@mail.ru (S.V.); chizhikvera@bk.ru (V.C.); 2 Federal State Budgetary Scientific Institution All-Russian Research Institute of Agricultural Biotechnology, 127550 Moscow, Russia; martynov.vik@gmail.com; 3 Bikovskaya Cucurbits Breeding Experimental Station—Branch of the Federal State Budgetary Scientific Institution “Federal Scientific Vegetable Center” (BCBES—Branch of the FSBSI FSVC), 400066 Volgograd, Russia; mari.kornilova.1978@mail.ru

**Keywords:** *Fusarium*, melon, morphology, pathogenicity, phylogenetic analysis, *F.* cf. *inflexum*, *F. brachygibbosum*

## Abstract

Fusarium wilt of melon, caused by *Fusarium* fungi, results in sizeable economic losses worldwide. In Russia, data on the species composition of the causative pathogens of this disease on melon are lacking. From 2022 to 2025, 19 *Fusarium* isolates from the Volgograd and Rostov regions were included in a study that included species identification using molecular phylogenetic analysis of the *tef1* and *rpb2* loci, morphological description, and pathogenicity assessment against the host plant and other members of the Cucurbitaceae family. Four *Fusarium* species were found to be involved in the pathogenesis of Fusarium wilt of melon in Russia: *F. clavus* (37% of the total number of isolates), *F. annulatum* (21%), *F.* cf. *inflexum* (21%), and *F. brachygibbosum* (21%). All identified species were isolated in the Volgograd Region, while only *F.* cf. *inflexum* and *F. brachygibbosum* were isolated in the Rostov Region. This study reports for the first time the high pathogenicity of *F.* cf. *inflexum* and *F. brachygibbosum* species associated with melon wilt. Morphological variability and different aggressiveness of isolates of the species *F. brachygibbosum* and *F. clavus*, isolated in the Volgograd (-V) and Rostov (-R) regions in different years, were observed. The isolate *F. brachygibbosum*-V showed high aggressiveness both at the sprout and seedling stages, while the isolate *F. brachygibbosum*-R was characterized by moderate aggressiveness only at the sprout stage. High pathogenicity of the species isolated from melons was established for other cucurbit crops. *F.* cf. *inflexum* was also pathogenic for watermelon and pumpkin, and *F. brachygibbosum* was pathogenic for pumpkin. The obtained data will have practical value for the development of biological control measures against *Fusarium* fungi and will be used in a melon breeding program for resistance to Fusarium wilt.

## 1. Introduction

Sale cropping of gourds, including melon (*Cucumis melo* L.), is one of the profitable and strategically important agricultural branches of the Russian economy [1,2]. The area under melon cultivation in farms of all categories in the Russian Federation currently amounts to 56.0 thousand hectares, with gross yields amounting to 785 thousand tons. National melon production is concentrated in three federal districts: Southern (45.4%), Volga (41.8%), and North Caucasus (8.5%) [3]. The main areas of the Southern Federal District, which includes the Volgograd and Rostov regions, are located in conditions without artificial irrigation. [4,5]. Along with specific weather conditions, the profitability of melon production in this region of Russia is affected by *Fusarium* infection. *Fusarium* fungi cause vascular wilt (FW), leaf spots, and fruit rot (BR) [6,7]. According to various researchers and farm producers, in Russia and worldwide, melon yield losses due to FW range from 30 to 100% [6,8,9,10,11], with economic damage of up to USD 22 million per season [12].

However, the harmfulness of *Fusarium* fungi manifests not only in crop yield losses, but also in their ability to synthesize and accumulate mycotoxins in plant tissues, which threaten human health [13,14,15]. *Fusarium* species exhibit great genetic and pathogenic variability. The structure of this genus is based on monophyletic clades called species complexes (SCs) [16]. Currently, the genus *Fusarium* counts more than 400 phylogenetic species distributed among 23 SCs [16,17]. An integrated approach is applied in order to unequivocally identify *Fusarium* species. This approach includes studying the morphological features of fungi [18,19] and phylogenetic analysis [20,21,22,23]. For phylogenetic differentiation of species groups, the combined use of marker sequences of genes encoding TEF-1α (translation elongation factor), RPB2, β-tubulin, and calmodulin is effective [24,25,26,27].

The economically important species *F. oxysporum* f. sp. *melonis* (FOM) is known as the prevailing causative agent of *Fusarium* wilt of melon [9,28]. Researchers around the world have conducted extensive studies to determine the racial composition of the *F. oxysporum* f. sp. *melonis* strains. Initially, depending on the host plant resistance genes, the pathogen was classified into races 0, 1, 2, and 1.2 [28,29,30]. Later, the most harmful race 1.2, which has now spread worldwide and is able to overcome known resistance genes, was divided into pathotypes 1.2y and 1.2w [10,29,30,31,32,33]. Pathotype 1.2y is known to cause leaf yellowing symptoms followed by plant death, while 1.2w causes wilting symptoms and death without prior yellowing symptoms [33].

According to recent scientific reports, along with the dominant species *F. oxysporum* f. sp. *melonis*, the following species are also involved in the pathogenesis of vascular wilt and root rot on melon: *F. commune*, *F. proliferatum*, *F. equiseti*, *F. delphinoides*, and *F. andiyazi*; *Neocosmospora falciformis* (=*F. falciforme*), *N. keratoplastica* (=*F. keratoplasticum*), and *F. petroliphilum* [34,35,36,37], belonging to a different species complex.

Taking into account that *Fusarium* chlamydospores can retain viability in soil for decades and fungicide use is often ineffective, the most cost-effective strategy for controlling *Fusarium* wilt is to breed cultivars resistant to species common in a particular production region [38,39,40]. Data on *Fusarium* species capable of forming the pathocomplex that causes *Fusarium* wilt on melon in Russia are absent to date, and without such knowledge, it is impossible to assess the harmfulness of these species and to target breeding for resistance to these species. The aim of this study was to identify the *Fusarium* species associated with melon wilt in Russia based on morphological and molecular characteristics and to assess their pathogenicity in accordance with Koch’s postulates.

## 2. Materials and Methods

### 2.1. Collection of Plant Material and Isolation of Fusarium Fungi

Melon plants with signs of vascular wilt and dry stem rot were collected in 2022 and 2025 in two southern regions of Russia: Volgograd (Bykovsky district) and Rostov (Tatsinsky district). In total, about 150 plants were collected in these regions. The Volgograd region has a severely continental and arid climate; the average annual precipitation is 314 mm; the average annual air temperature over a long period is 11.4 °C; in the summer months (June–August), temperatures range from +22 °C to +38 °C. The Rostov region has a humid continental climate with an average annual precipitation of 408 mm. The average annual temperature over a long period is 13 °C, and during the summer months (June–August) it ranges from 25 °C to 29 °C.

In order to obtain fungal isolates, plant tissues were cut into approximately 1 cm (usually 3–5 mm) long segments at the margin of the disease affected and healthy areas, surface sterilized in 70% ethanol, rinsed in sterile water, dried, and placed on potato dextrose agar PDA (20 g dextrose, 20 g agar, and the broth from 250 g white potatoes made up to 1 L with tap water). After incubation at 25 °C, supposed *Fusarium* colonies were transferred to new (fresh) potato dextrose agar (PDA) and synthetic nutrient agar SNA (KH_2_PO_4_—1 g, KNO_3_—1 g, MgSO_4_·7H_2_O—0.5 g, KCl—0.5 g, Glucose—0.2 g, Sucrose—0.2 g, Agar—20 g) media to obtain a pure culture of isolates and then single-spore colonies.

A total of 19 *Fusarium* isolates collected from melon plants with symptoms of systemic disease were tested for pathogenicity to confirm Koch’s postulates and included in the morphological characterization study and phylogenetic analysis (Table 1).

### 2.2. DNA Isolation and PCR

DNA isolation was carried out using the DNeasy Plant Pro Kit (QI-AGEN, Hilden, Germany). The primers used and the PCR conditions were the same as described previously [41,42]. In order to determine the species of the analyzed samples, we used GenBank NCBI [43] and FUSARIOID-ID database—Food, Fibre & Health [44,45].

### 2.3. Phylogenetic Analysis

Phylogenetic analysis involved 27 nucleotide sequences. Evolutionary analyses were conducted in Mega12 software using the UPGMA method [45].

### 2.4. Macro- and Micromorphology of Fusarium Isolates

In order to determine the macro- and micromorphological characteristics of *Fusarium* monospore cultures, isolates were grown for 7 days at 25 °C on potato dextrose agar (PDA), Czapek-Dox, and synthetic nutrient agar (SNA) media. Colony morphology, pigmentation, and fungal growth rate were assessed daily, recording the time at which growth began. The diameter of each colony was measured in two perpendicular directions three times. Microscopic characteristics of monospore cultures were examined and recorded using a Zeiss Axio Lab A1 microscope (ZEISS, Jena, Germany) and ADF software (version x64, 4.11.21522.20221011). For each isolate, at least 40 microstructures (conidia, chlamydospores) were measured. Based on the obtained data, the taxonomic status of the *Fusarium* fungi was determined in accordance with identification guides and scientific publications [19,46,47,48].

### 2.5. Assessment of Pathogenic Properties of Fusarium Isolates for Cucurbitaceae Crops at Different Stages of Plant Development

*Fusarium* isolates were tested for pathogenicity to confirm Koch’s postulates. The specificity of these isolates to infect melon host plants at various stages of ontogenesis was studied, as well as their ability to cause symptoms of damage on other *Cucurbitaceae* crops (pumpkin, watermelon, cucumber) at an early stage of development.

#### 2.5.1. Pathogenicity Test at the Sprout Stage

The study of the pathogenic properties of *Fusarium* isolates at the sprout stage was carried out according to a modified method [49,50]. Testing was carried out on the following plant material: four melon lineages with contrasting resistance levels under natural infection conditions in the Volgograd region (susceptible line C-IM-10, resistant line C-IM-6, moderately susceptible lines C-IM-7 and C-IM-1); three pumpkin lineages (Cp-IM-24, Cp-IM-29, Cp-IM-31), three watermelon lineages (Cl-IM-09, Cl-IM-55, Cl-IM-19); and three cucumber lineages (Cl-IM-09, Cl-IM-55, Cl-IM-19) bred by FSBSI FSVC and BCBES—branch of the FSBSI FSVC. Visually asymptomatic seeds of the analyzed *Cucurbitaceae* crops were sterilized in a 2% NaOCl solution for 10 min, rinsed three times in sterile water, and dried. To prevent latent infection, the seeds were pre-germinated in a humid chamber until a healthy root at least half the seed’s length emerged.

To prepare the inoculum, spore suspensions of 7-day-old colonies of *Fusarium* isolates, previously grown on PDA at 25 °C, were used. The concentration was 10^6^ spores/mL. Briefly, 250 mL of moistened sterile perlite was added to the bottom of a plastic container (volume—1200 mL, length—18 cm, width—14 cm, depth—10 cm), then 60 mL of spore suspension was added. Cucurbitaceae seeds were laid out, covered with a 500 mL layer of perlite on top, and the container was tightly closed with a lid to maintain humidity at 85–90%. In each container, 15 seeds of each line of pumpkin crops were sown in triplicate (n = 3). The control was perlite with sterile water.

The seeds were germinated in the dark for the first three days. After germination, the containers with the sprouts were incubated in a growth chamber at 65% relative humidity, 27 °C, and an 18 h/6 h (day/night) lighting schedule. These parameters were chosen to closely approximate the climatic conditions of southern Russia during the period of epiphytotics in cucurbits crops.

Observations of sprout development and manifestation of damage symptoms were carried out dynamically (7, 14, and 21 dpi), and the disease severity index (DSI) was calculated.

**Figure 1:**
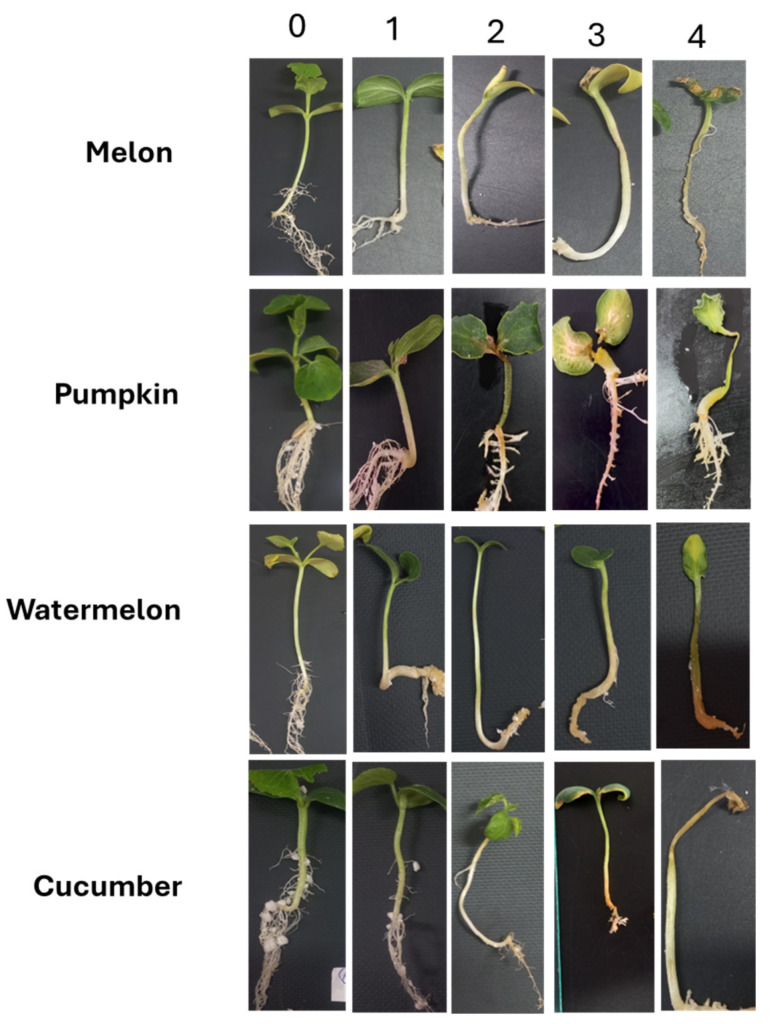
Scale for assessing symptoms of *Cucurbitaceae* sprout affection when infected with *Fusarium* isolates.

The assessment was performed using a 5-point scale [49], slightly modified for this study (Figure 1):

0—no symptoms;

1 point—taproot slightly brown, adventitious roots, and vegetative part without symptoms of damage;

2 points—taproot brown, adventitious roots with visible damage, vegetative part growth limited, and slight wilting;

3 points—taproot completely brown, adventitious roots poorly developed, stem growth 50% slower than control, severe wilting;

4 points—taproot dark brown, completely necrotic or may not develop at all, adventitious roots absent, plants completely necrotic or wilted.

At the end of the experiment, root length and stem height of sprouts were measured in both experimental and control treatments. The effect of the studied *Fusarium* isolates on sprout biometric parameters was assessed using the effect of action (EA), calculated using the formula:

$$Effect\;of\;action=\frac{Indicator\;in\;the\;experimental\;version-Indicator\;in\;the\;control\;version}{Indicator\;in\;the\;control\;version}\times 100$$

A negative EA value indicates inhibition, whereas a positive EA value indicates stimulation of the studied parameters after infection relative to the control.

Based on the disease severity index and impact on seedling growth and development, fungal isolates were classified as:

• Non-pathogenic (n/p)—plants are affected by 0–0.4 points.
• Weakly aggressive (WA)—plants are affected by 0.5–0.8 points.
• Moderately aggressive (MA)—50% of lines are affected by 0.9–1.8 points.
• Highly aggressive (HA)—more than 75% of lines are affected by 1.8–4.0 points.

#### 2.5.2. Study of the Aggressiveness of Fusarium Isolates Against Melon at the Stage of 21-Day-Old Seedlings (Phase of the First Pair of True Leaves)

In this experiment, the same four melon lines (C-IM-7, C-IM-1, C-IM-10, and C-IM-6) were used in triplicate (n = 3), with ten plants of each line per replication. The inoculum preparation of 20 *Fusarium* isolates for infection was described above. Seedlings were grown in plastic trays with a compartment volume of 150 mL in a peat–perlite mixture at a ratio of 3:1 in a growth chamber under 18 h/6 h (day/night) illumination.

Artificial inoculation of 21-day-old seedlings was carried out according to a previously known protocol [9,50]. Seedlings were carefully removed from the soil, the roots were rinsed with tap water, trimmed to 1 cm, and immersed for 30 min in a spore suspension (10^6^ spores/mL) of the corresponding isolate. Infected seedlings were planted in 1000 mL pots containing a sterile peat–perlite mixture in a 3:1 ratio. Incubation was carried out in a controlled growth chamber at 27 °C with the same photoperiod for three weeks. Seedling development and damage symptoms were observed dynamically at 7, 14, and 21 dpi. The severity of damage was assessed using a previously described scale [9]. The disease severity index (DSI) and the effect of action (EA) were calculated for the experimental variants relative to the control.

In order to further study the level of aggressiveness of the four most aggressive strains of different *Fusarium* species—FV-C-3 (*F. annulatum* (FFSC)), FR-C-28 (*F.* cf. *inflexum* (FOSC)), FV-C-11 (*F. brachygibbosum* (FSAMSC)), and FV-C-12 (*F. clavus* (FIESC))—against melon plants, we used another 29 melon lines bred by BCBES, a branch of the FSBSI FSVC.

### 2.6. Statistical Processing

Experimental data analysis and statistical evaluation were performed using Microsoft Excel 2016 for Windows 10 and Statistical 7.0 software. Duncan’s test with a 95% probability (*p* ≤ 0.05) was used to determine the significance of differences in the aggressiveness of *Fusarium* strains. The influence of factors on the severity of damage of different melon lines was assessed using analysis of variance.

## 3. Results

### 3.1. Symptoms of Fusarium Wilt in the Field

**Figure 2:**
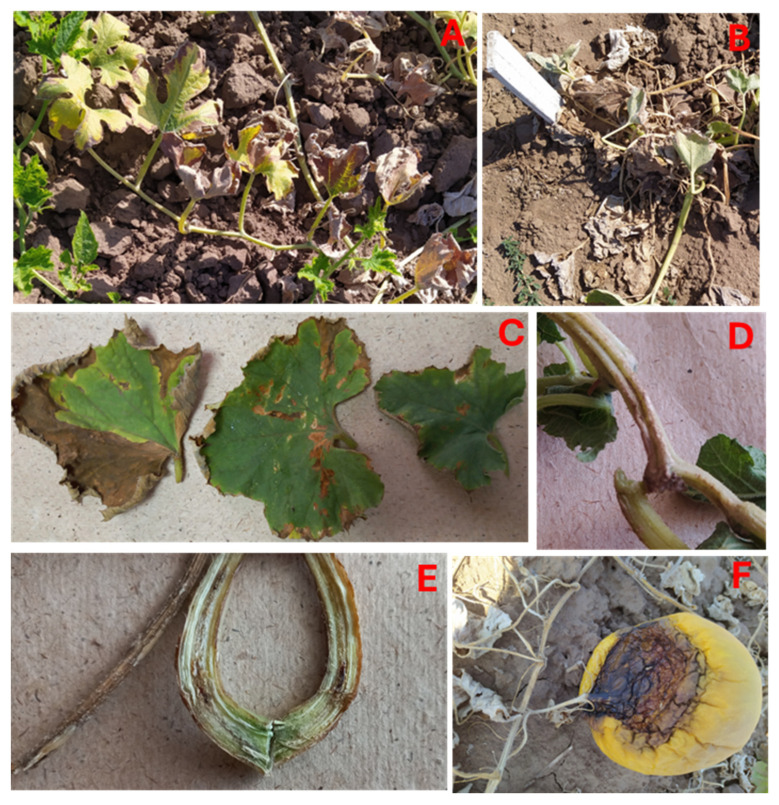
Symptoms of *Fusarium* disease on melon in the southern regions of Russia: typical symptoms of wilting (**A**,**B**), necrosis of leaves (**C**), and the vascular system (**D**,**E**) in the Volgograd and Rostov regions; (**F**) fruit lesion in the Volgograd region.

Phytopathological monitoring in the Volgograd (2022, 2025) and Rostov (2025) regions revealed that the pattern of symptom manifestation on melon plants in the early stages of infection was similar in these regions (Figure 2A–E). The first signs of the disease appeared during the flowering phase, beginning with stem chlorosis, followed by loss of turgor, wilting, and eventually complete drying up in case of susceptible varieties (Figure 2A,B). When dissecting the internodes, necrosis of the vascular tissue was observed (Figure 2D,E).

During this period, necrosis began to develop in the peripheral parts of the leaves, gradually spreading across the entire lamina. In some cases, necrotic spots of varying sizes formed in the leaf parenchyma between the veins (Figure 2C). A distinctive feature of Fusarium wilt in the Volgograd region was the infection not only of the vascular system but also of the reproductive organs. Necrosis from the peduncle spread to the upper part of the fruit, causing browning and dry rot (Figure 2F).

Thus, 19 *Fusarium* isolates were obtained from different parts of the affected melon plants with symptoms of Fusarium wilt and rot. These isolates were studied for species identification (molecular, phylogenetic, and morphological) and for their phytopathogenic properties.

### 3.2. Phylogenetic Analysis of Fungi Fusarium

**Figure 3:**
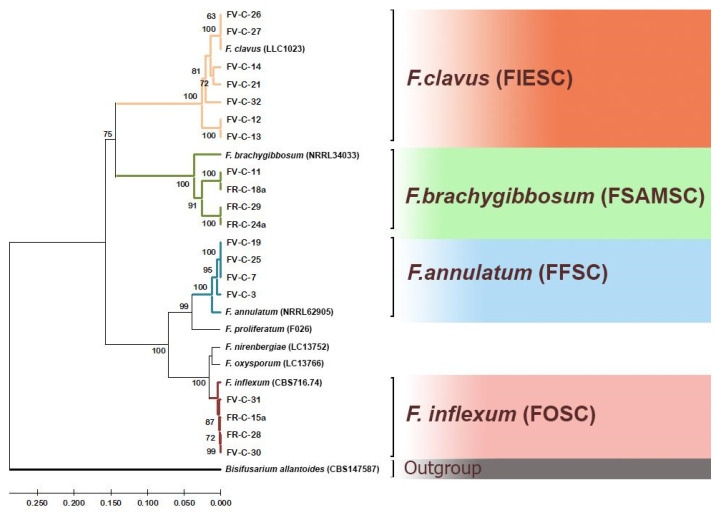
Phylogenetic tree based on DNA sequences of *tef1* and *rpb2* loci of *Fusarium* strains. The bootstrap values (1000 replicates) are shown at the nodes. The sequences of *Bisifusarium allantoides* strain CBS 147587 are an outgroup.

To determine the species membership of the studied isolates, the nucleotide sequences of the tef1 and rpb2 loci in these isolates were compared with reference sequences of these loci from the NCBI database and the supervised FUSARIOID-ID database. The resulting dendrogram is shown in Figure 3. In this dendrogram, all sequences of the analyzed loci clustered with reference sequences for specific species, allowing for unambiguous identification of the species of the studied isolates.

Strains FV-C-12, FV-C-13, FV-C-14, FV-C-21, FV-C-26, FV-C-27, and FV-C-32 belong to the species *F. clavus*, *F. incarnatum-equiseti* species complex (FIESC). Strains FV-C-11, FR-C-18a, FR-C-24a, and FR-C-29a belong to the species *F. brachygibbosum*, *F. sambucinum* species complex (FSAMSC). Strains FV-C-3, FV-C-7, FV-C-19, and FV-C-25 belong to the species *F. annulatum* of the *F. fujikuroi* species complex (FFSC, previously GFSC). Strains FR-C-15a, FR-C-28, FV-C-30, and FV-C-31 belong to the species *F.* cf. *inflexum*, *F. oxysporum* species complex (FOSC). Thus, we identified species *F. clavus*, *F. brachygibbosum*, *F. annulatum*, and *F.* cf. *inflexum* as causative agents causing melon wilt in Russia based on molecular characteristics. Nucleotide sequences have been deposited in the NCBI database (PX512066–PX512084).

For the final and accurate species determination of the studied *Fusarium* strains, we combined the results of molecular phylogenetic and macro- and micromorphological analysis.

Thus, four *Fusarium* species from different species complexes were identified as part of the pathocomplex of causal agents of Fusarium wilt of melon in the Volgograd and Rostov regions: *F. annulatum* (FFSC), *F.* cf. *inflexum* (FOSC), *F. brachygibbosum* (FSAMSC), and *F. clavus* (FIESC). All identified species were involved in the pathogenesis of Fusarium wilt of melon plants in the Volgograd region; only their percentages varied. In the Rostov region, the species complex isolated from the analyzed plants was less diverse and was represented by *F.* cf. *inflexum* (FOSC) and *F. brachygibbosum* (FSAMSC). At the same time, *F. clavus* dominated the overall structure of the analyzed pathocomplex (37% of the total number of isolates), while the percentages of the remaining species were equal, accounting for 21% each.

### 3.3. Morphological Characteristics of Fusarium Species Associated with Fusarium Wilt of Melon

**Figure 4:**
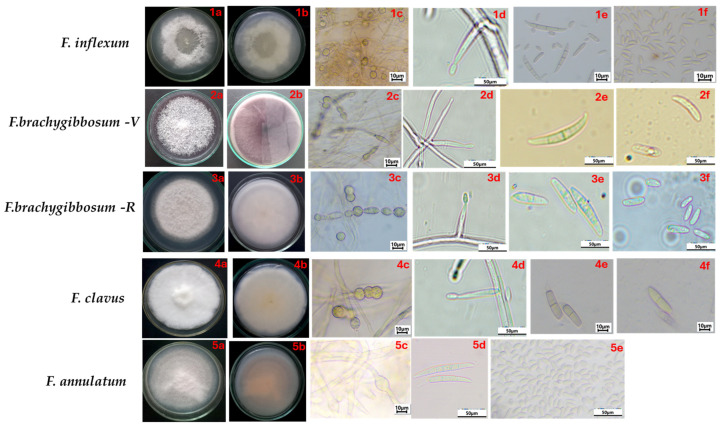
Cultural and micromorphological characteristics of *Fusarium* species isolated from melon (PDA at 25 °C, 16 h light/8 h dark, 7 days). Scale bars (**1c**,**1e**,**1f**,**2c**,**3c**,**4c**,**4e**,**4f**,**5c**) = 10 μm; and (**1d**,**2d**–**2f**,**3d**–**3f**,**4d**,**5d**,**5e**) = 50 μm. *F.* cf. *inflexum*: (**1a**,**1b**) obverse and reverse; (**1c**) chlamydospores; (**1d**) monophialides, (**1e**) macroconidia; (**1f**) microconidia. *F. brachygibbosum-*V: (**2a**,**2b**) obverse and reverse; (**2c**) chlamydospores; (**2d**) monophialides and polyphialides, (**2e**) macroconidia; (**2f**) microconidia. *F. brachygibbosum-*R: (**3a**,**b**) obverse and reverse; (**3c**) chlamydospores; (**3d**) monophialides, (**3e**) macroconidia; (**3f**) microconidia. *F. clavus*: (**4a**,**4b**) obverse and reverse; (**4c**) chlamydospores; (**4d**) monophialides, (**4e**) macroconidia; (**4f**) microconidia. *F. annulatum*: (**5a**,**5b**) obverse and reverse; (**5c**) chlamydospores; (**5d**) macroconidia; (**5e**) microconidia.

The descriptions of colonies and micromorphological characteristics of fungi belonging to *Fusarium* species causing wilt of melon plants are presented in Table 2 and Figure 4. Isolates of all studied species produced macro- and microconidia of various shapes and sizes; chlamydospores of various shapes and sizes, mainly intercalary solitary, in pairs or chains; conidiogenous cells were not found, only in *F. annulatum*. The morphological characteristics of *F. clavus* and *F. annulatum* were consistent with the original descriptions available in the literature. For *F. brachygibbosum*, differences in cultural and morphological characteristics were found, depending on the geographic region of origin. Particularly, the species *F. brachygibbosum*-V (Volgograd region) on PDA medium typically formed colonies with a red pigment in the center and varying shades of purple at the edges. Macroconidia were long, variably curved, with apical cells rounded and basal cells with a typical stalk-like shape. Microconidia were slightly curved. Chlamydospores were slightly elongated, and conidiogenous cells were represented by mono- and polyphialides. The species *F. brachygibbosum*-R (Rostov region) formed pink colonies with powder-like mycelium on PDA medium. Macroconidia are straighter, with a slightly expanded central cell, and are smaller than those isolated in the Volgograd region. Microconidia have an oval shape. It produces only monophialides.

### 3.4. Study of Pathogenic Properties of Fusarium Isolates for Melon at the Sprout Stage

The outcome of melon sprout infection with isolates of various *Fusarium* species depended on a number of factors: host plant resistance (variety specificity), pathogen species, and origin (region, year, organ) (Table 3). The severity of damage and symptom manifestation in melon lines after artificial inoculation corresponded to their field resistance level: melon line C-IM-6 demonstrated resistance to all analyzed *Fusarium* species (DSI = 0), while the remaining lines were susceptible to varying degrees (DSI = 1.12–1.28).

A significantly high pathogenicity level (*p* ≤ 0.05) was established for *F.* cf. *inflexum* isolates: the average damage score for all lines was DSI = 2.01 ± 0.85. Three isolates of this species (FR-C-28, FR-C-15a, and FV-C-30), isolated from the stems and roots of affected plants in both regions, were highly aggressive for most of the analyzed lines (DSI—from 2.4 ± 0.61 to 4.0).

All analyzed *F. annulatum* isolates demonstrated low pathogenicity against melon plants (DSI = 0–0.45), and the average damage score in the experimental group of all lines was statistically indistinguishable from the control group. Only a few seedlings showed mild symptoms of wilting by the end of the experiment. A different trend was observed for *F. brachygibbosum* and *F. clavus* isolates. Isolates of these species caused varying degrees of sprout wilting depending on the resistance of the melon lines.

In case of wilt, the DSI for *F. brachygibbosum* isolates ranged from 0.03 for isolate FR-C-24a (isolated from leaves in the Rostov region) to 1.82 ± 1.15 for isolate FV-C-11 (isolated from stems in the Volgograd region). It was also established that the degree of aggressiveness of isolate FV-C-11 was significantly higher than that of isolate FR-C-29, also isolated from stems in the Rostov region. When infected with isolate FV-C-11 (Volgograd region), only the resistant line C-IM-6 exhibited high resistance; the other three lines were highly susceptible (DSI = 2.3–2.6). When infected with isolate FR-C-29 (Rostov region), the degree of aggressiveness varied from a minimum of 0.35 ± 0.13 (C-IM-1) to an average of 1.55 ± 0.26 (C-IM-7). The DSI of these isolates averaged 1.82 ± 1.15 and 0.68 ± 1.35 across all lines, respectively.

For the prevailing *F. clavus* isolates found only in the Volgograd region, a change in pathogenicity was observed depending on the year of study. Of the three isolates collected in 2022, only one—FV-C-12—exhibited high aggressiveness (DSI = 2.0–2.9); the other two, FV-C-13 and FV-C-14, were moderately aggressive (DSI = 1.0). All four *F. clavus* isolates collected in 2025 exhibited low pathogenicity against test lines (DSI = 0–0.65).

An ANOVA of the aggressiveness of all isolates obtained from stems and leaves revealed the strongest effect of fungal species on infection outcome (34.0% of the variance), as well as a significant effect of their localization (the affected plant organ), which accounted for 7.3% of the total variance. However, the variance of their interaction was insignificant (2.6%).

The morphological and molecular characteristics of the isolates re-isolated from artificially inoculated plants and the original isolates were identical, which confirmed Koch’s postulates.

### 3.5. Study of Pathogenic Properties of Fusarium Fungi Against Melon at the Stage of the First Pair of True Leaves

When melon plants at the stage of the first pair of true leaves were infected with *Fusarium* isolates, either a similar or a different response of seedlings was observed compared to the response of sprouts (Table 4).

The same trend of low aggressiveness was observed for *F. annulatum* isolates: they did not infect seedlings of any melon line studied (DSI = 0–0.45), and by the end of the final survey, plants from the experimental treatments were indistinguishable from the control plants. Three *F.* cf. *inflexum* isolates were characterized by high aggressiveness on average (DSI = 1.5), and one isolate was moderately aggressive (DSI = 0.75), which was also observed during infection at the sprout stage.

Changes in the response of the analyzed melon lines to infection with *F.* cf. *inflexum* isolates were observed. Line C-IM-6, resistant at the sprout stage, demonstrated sensitivity to infection by highly aggressive isolates of this species, and by the time of final survey, wilting of plants was observed with a DSI of 0.45 ± 0.28. Conversely, seedlings of line C-IM-1 were more resistant at this stage of ontogenesis compared to the sprout stage (DSI = 0.7 ± 0.41).

The ability to strongly affect plants of the studied lines at the seedling stage was retained only for one isolate of *F. brachygibbosum* (FV-C-11), isolated in the Volgograd region: for more susceptible lines (C-IM-7 and C-IM-10) DSI = 2.7 ± 0.47 and 3.0 ± 0.69; for more resistant lines (C-IM-6 and C-IM-1)–DSI = 0.23 ± 0.26 and 0.48 ± 0.11. The trend was also maintained for the *F. clavus* isolate FV-C-12, characterized by the highest aggressiveness at both stages of plant development in comparison with other *Fusarium* species. According to the average data, its DSI at the seedling stage was 1.78. The remaining analyzed isolates at the seedling stage were nonpathogenic or showed weak aggressiveness against melon lines.

### 3.6. Study of Aggressiveness of Fusarium Species Against Melon at the Stage of First Pair of True Leaves

The most aggressive isolates of each identified *Fusarium* species were included in the next stage of research by infecting genetically diverse host plant material at the seedling stage. This allows for the assessment of their prospective harmfulness to this crop. A total of four *Fusarium* isolates and 29 melon breeding lines with varying levels of field resistance were included in this experiment.

A study of the disease development dynamics revealed differences between these isolates in terms of the onset of initial symptoms (Table 5). When infected with *F.* cf. *inflexum* and *F. brachygibbosum*, plants had a similar type and pattern of symptomatology: the first signs of wilting of highly susceptible melon lines were observed already on the 7th day, and complete death of seedlings on the 14th day. By the time of the final survey on the 21st day, a significant (*p* ≤ 0.05) broad virulence of *F.* cf. *inflexum* and *F. brachygibbosum* against the tested lines was revealed. During the experiment, most of them (75.8% and 69.5%) showed high DSI when inoculated with these species (2.94 and 2.44, respectively), and the inhibition of the root system development (EA = −38.5 … −16.7%) and the above-ground part of the seedlings (EA = −16.7 … −15.5%).

The pattern of symptom development following infection with *F. clavus* differed somewhat from that observed in the species described above. The severity of plant damage in 20% of lines was lower than that observed with *F.* cf. *inflexum* and *F. brachygibbosum* (DSI = 1.96). Moreover, symptoms appeared within 10–12 days as basal stem rot, resulting in rapid seedling death in 8% of lines.

No clear visual symptoms were observed during the experiment when plants were inoculated with the *F. annulatum* species. Although some plants from more sensitive lines (2.1% of the total studied) showed mild signs of effect, the differences compared to the control plants were insignificant across all plants analyzed.

### 3.7. Study of the Pathogenicity of Fusarium Species Against Cucurbitaceae Crops at the Sprout Stage

**Figure 5:**
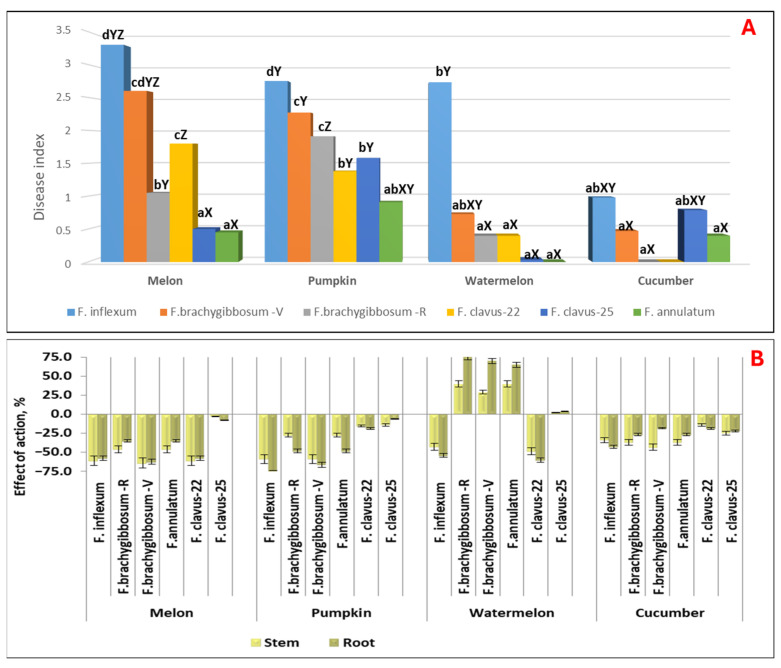
Pathogenicity of isolates of different *Fusarium* species in terms of the disease index (**A**) and the effects on biometric parameters (**B**) for Cucurbitaceae crops (14 dpi). Note: For comparability of results, the average data for melon from Table 2 are presented. a–d—values with the same lowercase letter are not significantly different for *Fusarium* isolates; X–Z—values with the same uppercase letter are not significantly different for Cucurbitaceae crops with a 95% probability according to Duncan’s test. The diagrams present the average values of the disease index for the set of all analyzed lines of each crop infected with the isolate.

**Figure 6:**
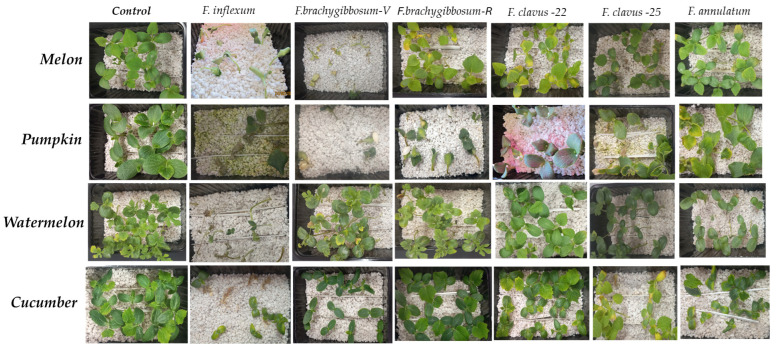
Effects of the activity of various *Fusarium* species on Cucurbitaceae (21 dpi).

The study on the pathogenicity of *Fusarium* species for Cucurbitaceae crops (pumpkin, watermelon, and cucumber) included isolates that demonstrated high aggressiveness against melon. These are representatives of the following species: *F.* cf. *inflexum* (FR-C-28); *F. brachygibbosum* (FV-C-11 and FR-C-29) from the Volgograd and Rostov regions, respectively; *F. clavus* (FV-C-12 and FV-C-21), isolated in 2022 and 2025, respectively; and *F. annulatum* (FV-C-3). Infection of pumpkin crops was carried out at the sprout stage, as it is the most sensitive phase. Cross-pathogenicity analysis of isolates of different *Fusarium species* isolated from melon revealed differences in their impact on the mean disease index (Figure 5A), their effects on root and stem growth (Figure 5B), and the specificity of symptoms on Cucurbitaceae sprouts (Figure 6). A common feature for all highly aggressive isolates against Cucurbitaceae crops is the initial appearance of chlorosis symptoms, followed by dry rot at 14 dpi, whereas on the host plant, they caused symptoms of wilting and damage to the vascular system (Figure 6). Moderately aggressive isolates caused varying degrees of chlorotic symptoms both on melon and other Cucurbitaceae crops.

A significantly high aggressiveness of the *F.* cf. *inflexum* species (*p* ≤ 0.05) was established against both the host plant and pumpkin (DSI = 2.75) and watermelon (DSI = 2.75) (Figure 5A). For these crops, strong inhibition of stem and root system development was noted: EA stem = −59.2 (for pumpkin) and −43.0 (for watermelon); EA root = −78.3 (for pumpkin) and −54.4 (for watermelon) (Figure 5B). The remaining analyzed *Fusarium* species showed weak pathogenicity for watermelon (*F. annulatum*, DSI = 0–0.73) with leaf chlorotic symptoms on sprout, or were nonpathogenic (DSI = 0–0.4). Moreover, if isolates of the species *F.* cf. *inflexum* and *F. clavus*-22 caused strong inhibition of the stem and root in watermelon sprout, then isolates of the species *F. brachygibbosum*, isolated from two regions, and *F. annulatum* caused statistically significant stimulation of biometric parameters (EA stem = 29.0–39.6 and EA root = 65.0–75.0, depending on the isolate).

Pumpkin was the most sensitive to all isolates. Statistically significant disease indices, different from the control, ranged from 0.91 to 1.58 for moderately aggressive isolates of *F. annulatum*, F. clavus-22, and *F. clavus*-25, to 1.91–2.75 for highly aggressive *F.* cf. *inflexum* and *F. brachygibbosum*.

Only the isolates *F.* cf. *inflexum* and *F. clavus*-25 exhibited moderate aggressiveness against cucumber, with statistically significant DSI values of 0.98 and 0.79, respectively. These isolates caused severe chlorosis of the sprouts and stunted growth, with 5 to 27% of seeds rotting and failing to germinate.

## 4. Discussion

Due to their scientific importance and economic harmfulness, *Fusarium* species are among the ten most important global pathogenic fungi for agricultural crops, including melon [9,51]. Currently, there is no information about the composition of the pathocomplex that causes *Fusarium* wilt of melon in Russia, although yield losses from this disease are increasing annually [1]. This study, using melon crops, was the first to examine the genetic diversity of *Fusarium* fungi that cause wilt in strategically important regions of Russia for this crop production. Molecular phylogeny, according to sequences of *tef1* and *rpb2* genes, and morphological assessment and analysis of the pathogenic properties in the pathocomplex revealed the presence of four *Fusarium* species from different species complexes—*F. annulatum* (FFSC), *F.* cf. *inflexum* (FOSC), *F. brachygibbosum* (FSAMSC), and *F. clavus* (FIESC). It was established that all identified species were involved in the pathogenesis of *Fusarium* wilt of melon plants in the Volgograd Region, while in the Rostov Region, only *F.* cf. *inflexum* (FOSC) and *F. brachygibbosum* (FSAMSC) were involved in this process. In the overall structure of the analyzed pathocomplex, *F. clavus* dominated (37% of the total isolates), while the remaining species were equally represented (21% each). According to other authors, in addition to the most common species, *F. oxysporum* f. sp. *melonis* (FOM), other representatives of the genera *Fusarium* and *Neocosmospora* can be causative agents of *Fusarium* wilt of melons, namely *F. petroliphilum*, *F. commune*, *F. proliferatum*, *F. equiseti*, *F. delphinoides*, *F. andiyazi*, *N. falciformis*, and *N. keratoplastica* [9,36,46]. Thus, none of the species associated with melon wilt identified in the present study had been described as causative agents of Fusarium wilt of melon previously.

In the present study, the high pathogenicity of the *F. oxysporum*-related species *F.* cf. *inflexum* from FOSC against the host plant was observed. Although many publications contain data on the biodiversity of this important species complex and its reclassification [52], reports on the study of *F. inflexum* are scarce, and publications do not describe the morphology of this species. In recent years, this species was isolated and showed aggressiveness in the pathogenesis of bean wilt [53], peanut leaf spot in China (*Arachis hypogaea*) [54], wilt and root rot of coniferous seedlings in the USA [55], and root and trunk rot of grapevine shoots in Brazil [56]. In our study, isolates of this species from the Volgograd and Rostov regions were highly aggressive in melon and had broad virulence when infected at the early stages of ontogenesis: at the sprout stage (DSI = 2.01 ± 0.85), seedlings in the phase of the first pair of true leaves (DSI = 2.94 ± 1.81).

*F. brachygibbosum* is known to infect a wide range of host plants, causing wilt and rot on Fabaceae crops [57], corn [58], and sunflower [59]. In Russia, this species was found on strawberries with symptoms of root rot [60]. In our studies, differences in cultural, morphological, and pathogenic characteristics were registered for isolates of this species from the Volgograd and Rostov regions. The isolate *F. brachygibbosum*-V (Volgograd region) formed colonies with a reddish-violet pigment in the stroma with a darker center, curved macroconidia, the basal cells of which had a typical stalk-like shape. Whereas colonies of the isolate *F. brachygibbosum*-R (Rostov region) had a pink pigment, the macroconidia were straighter and smaller in size compared to isolates obtained in the Volgograd region. These isolates also showed statistically significant differences in their levels of aggressiveness. The isolate *F. brachygibbosum*-V demonstrated high aggressiveness at both the sprout and seedling stages; the isolate from the Rostov region exhibited moderate aggressiveness only at the sprout stage. The broad range of variability of the morphological characteristics of *F. brachygibbosum* isolates was also observed in the watermelon culture, where the authors identified four different morphotypes of this species in one region [61].

Another species we studied, *F. clavus* (FIESC), was described in the literature as an endophyte in melon, the colonization of which mitigated the harmful effects of salt stress in seedlings [62]. Previously, the high pathogenicity of this species was established for pepper in the southern regions of Russia [63], for tomatoes in Italy [64], for date palms in Tunisia [47], and for potatoes in Algeria [65]. In our study, *F. clavus* isolates collected in 2022 showed high and medium levels of aggressiveness when inoculated onto sprouts and seedlings of different melon lines with varying levels of resistance, while isolates collected in 2025 were non-pathogenic or weakly pathogenic against the host plant.

High morphological variability, genetic intraspecific diversity, and changes in the pathogenicity of *Fusarium* fungi depending on temperature and soil composition [66], geographical location, and the year of obtaining isolates from affected plants have also been described by other authors [55,63]. According to some researchers, these differences may be due to additional virulence factors [67,68], horizontal transfer of genetic material through conidial anastomosis tubes [69,70], and prolonged cultivation on artificial nutrient media [71]. Therefore, in future studies of the species composition of melon wilting, we will expand phylogenetic research by incorporating new methodological approaches, increasing the number of agroclimatic zones of Russia, and including a wider range of melon varieties of different origins.

Cultivating gourds requires large areas of land; therefore, in Russia, a four-field crop rotation system is used, involving watermelon, melon, pumpkin, and fallow land. This cultivation technology can lead to a decrease in the number of beneficial microorganisms in the soil [72] and an increase in the aggressiveness of phytopathogens against other members of the Cucurbitaceae family [46,73,74,75]. In this study, when assessing cross-pathogenicity for other cucurbit crops (watermelon, pumpkin, cucumber), it was found that a highly aggressive *F.* cf. *inflexum* isolate, isolated from melon, is capable of infecting watermelon and pumpkin sprouts; and the *F. brachygibbosum* isolate infects pumpkin sprouts, primarily causing root and stem rot. The isolate *F. clavus*-22 caused statistically significant inhibition of root and stem development in watermelon, pumpkin, and cucumber sprouts without typical symptoms of rot. This suggests the possible involvement of the isolated *Fusarium* species in the pathogenesis of root rot and tracheomycotic wilting in other cucurbit crops, which requires further research in this area.

To assess the pathogenicity of *Fusarium* fungi for plants, including Cucurbitaceae, a method of placing seeds in Petri dishes directly on the surface of the fungal culture is used [49,50,76,77,78,79], which is quite problematic when working with large-seeded cucurbit crops. Therefore, in this study, for the initial rapid assessment of the pathogenicity and cross-pathogenicity of a large number of isolates against these crops, we used a quickly reproducible method of inoculation at the sprout stage by sowing germinated seeds in an infected substrate (in our modification, sterile perlite). Infecting the root system of seedlings makes it possible to identify among the isolated aggressive isolates the true causative agents of tracheomycotic Fusarium wilt, which are specific to a particular host plant and capable of penetrating its vascular system. Since many pathogenic Fusarium species, when penetrating root tissue, cause only symptoms of localized root rot, which does not always lead to wilting of the plants [80].

This study provides new information about the diversity, pathogenicity, and species identification challenges of *Fusarium* fungi causing tracheomycotic wilt of melons in Russia. It reports for the first time the high pathogenicity for melon crops of *F.* cf. *inflexum* and *F. brachygibbosum*, associated with this disease. The presented results are part of breeding and immunological studies aimed at creating melon varieties resistant to Fusarium wilt.

## 5. Conclusions

The study yielded new insights into the diversity and pathogenicity of *Fusarium* fungi that cause melon wilt in strategically important melon-producing regions of Russia. Molecular phylogeny using phylogenetic analysis of the *tef1* and *rpb2* loci identified four *Fusarium* species: *F. annulatum* (FFSC), *F.* cf. *inflexum* (FOSC), *F. brachygibbosum* (FSAMSC), and *F. clavus* (FIESC). High pathogenicity of *F.* cf. *inflexum* and *F. brachygibbosum*, associated with *Fusarium* wilt of melon, is reported for the first time. All identified species were involved in the pathogenesis of melon wilt in the Volgograd region, while only *F.* cf. *inflexum* and *F. brachygibbosum* were involved in the pathogenesis of this disease in the Rostov region. Among the total number of analyzed isolates, *F. clavus* was the prevailing species (37%), while the remaining species were represented in equal percentages (21% each). Morphological variability and varying levels of aggressiveness were observed in the case of *F. brachygibbosum* and *F. clavus*, isolated from different regions in different years. High pathogenicity of species isolated from melon plants was established for other cucurbit crops. Specifically, *F.* cf. *inflexum* was pathogenic for watermelon and pumpkin, and *F. brachygibbosum* was pathogenic for pumpkin. This fact requires special attention when planning agricultural practices, including crop rotation. The obtained data will be of practical value for developing measures to combat *Fusarium* fungi that cause melon wilt. Furthermore, data on the species composition and aggressive isolates will be used in a melon breeding program for resistance to Fusarium wilt.

## Figures and Tables

**Table 1:** *Fusarium* isolates found on melon plants and included in the study.

Strain	*Fusarium* Species	Origin	Substrate	Year
FV-C-3	*F. annulatum* (FFSC)	Volgograd	stem	2022
FV-C-7	*F. annulatum* (FFSC)	Volgograd	root	2022
FV-C-19	*F. annulatum* (FFSC)	Volgograd	leaf	2025
FV-C-25	*F. annulatum* (FFSC)	Volgograd	leaf	2025
FR-C-28	*F.* cf. *inflexum* (FOSC)	Rostov	stem	2025
FR-C-15a	*F.* cf. *inflexum* (FOSC)	Volgograd	stem	2025
FV-C-30	*F.* cf. *inflexum* (FOSC)	Volgograd	root	2025
FV-C-31	*F.* cf. *inflexum* (FOSC)	Volgograd	leaf	2025
FV-C-11	*F. brachygibbosum* (FSAMSC)	Volgograd	stem	2022
FR-C-18a	*F. brachygibbosum* (FSAMSC)	Volgograd	leaf	2022
FR-C-29	*F. brachygibbosum* (FSAMSC)	Rostov	stem	2025
FR-C-24a	*F. brachygibbosum* (FSAMSC)	Rostov	leaf	2025
FV-C-12	*F. clavus* (FIESC)	Volgograd	stem	2022
FV-C-13	*F. clavus* (FIESC)	Volgograd	leaf	2022
FV-C-14	*F. clavus* (FIESC)	Volgograd	leaf	2022
FV-C-21	*F. clavus* (FIESC)	Volgograd	fruit	2025
FV-C-26	*F. clavus* (FIESC)	Volgograd	stem	2025
FV-C-27	*F. clavus* (FIESC)	Volgograd	stem	2025
FV-C-32	*F. clavus* (FIESC)	Volgograd	stem	2025

Note: FFSC—*F. fujikuroi* species complex; FOSC—*F. oxysporum* species complex; FSAMSC—*F. sambucinum* species complex; FIESC—*F. incarnatum-equiseti* species complex.

**Table 2:** Cultural and micromorphological features of *Fusarium* strains isolated from melon.

Characteristic	*F.* cf. *inflexum* (FR-C-28)	*F. brachygibbosum-V* (FV-C-11)	*F. brachygibbosum-R* (FR-C-29)	*F. clavus* (FV-C-12)	*F. annulatum* (FV-C-3)
Colony:					
growth rate on PDA, mm/day ± SE	10.82 ± 0.03	10.60 ± 0.19	8.18 ± 0.14	9.55 ± 0.14	7.57 ± 0.06
mycelium	White, sparse, heterogeneous	White, felt	White, felt	White, dense, uniform,	White, loose, heterogeneous
pigment	lemon on day 14	purple on day 7	pink on day 18	yellowish on days 18–20	cream on day 12
type of conidiophores	monophialides	polyphialides and monophialides	monophialides	monophialides	not found
Microconidia:					
size, µm	9.2 ± 1.8 × 3.7 ± 0.5	11.5 ± 4.1 × 3.4 ± 0.7	9.65 ± 1.2 × 3.4 ± 0.4	4.9 ± 0.6 × 3.4 ± 0.2	5.84 ± 1.8 × 2.6 ± 0.4
septation	0	0	1	0–1	1
shape	oval or round	oval	slightly curved	oval to slightly curved	oval
Macroconidia:					
size, µm	25.9 ± 5.3 × 3.9 ± 0.9	23.0 ± 4.4 × 4.5 ± 0.7	20.2 ± 5.5 × 3.1 ± 0.5	14.1 ± 0.3 × 2.7 ± 0.1	25.8 ± 0.9 × 3.9 ± 0.2
septation	3	3–4	2–3	2–3	2–3
shape	slightly curved	slightly curved	straight	fusiform or slightly curved	almost straight
Chlamydospores:					
size, µm	7.4 ± 1.3 × 7.4 ± 0.9	10.2 ± 1.1 × 7.3 ± 1.2	9.6 ± 0.8 × 9.6 ± 0.9	10.4 ± 0.9 × 10.1 ± 1.1	10.6 ± 0.1 × 10.6 ± 0.9
shape	globose	globose or oblong	globose	globose	globose
abundance	in abundance, single or in pairs	more often in pairs	single, in pairs, in chains	in abundance, 3–4 in chains	single

**Table 3:** Aggressiveness of *Fusarium* isolates against melon sprouts (14 dpi).

Strain	Type	Aggressiveness Degree *	Disease Index *					
			Variety				Average for Isolate	Average for *Fusarium* Species
			C-IM-7	C-IM-1	C-IM-10	C-IM-6		
	Control		0 aX	0 aX	0 aX	0 aX	0 a	0 a
FV-C-3	*F. annulatum* (FFSC)	WA	0.45 ± 0.28 aX	0.35 ± 0.63 aX	0.3 ± 0.95 aX	0 aX	0.28 ± 0.68 a	0.08 ± 0.14 a
FV-C-7	*F. annulatum* (FFSC)	n/p	0 aX	0 aX	0 aX	0 aX	0 a	
FV-C-19	*F. annulatum* (FFSC)	WA	0.21 ± 0.03 aX	0 aX	0 aX	0 aX	0.05 a	
FV-C-25	*F. annulatum* (FFSC)	WA	0.38 ± 0.02 aX	0 aX	0 aX	0 aX	0.10 a	
FR-C-28	*F.* cf. *inflexum* (FOSC)	HA	2.7 ± 0.67 cZ	3.3 ± 1.16 cdZ	4 dZ	0 aX	2.50 ± 0.83 d	2.01 ± 0.85 d
FR-C-15a	*F.* cf. *inflexum* (FOSC)	HA	2.6 ± 0.73 cZ	3.0 ± 1.39 cdZ	3.67 ± 0.95 dZ	0 aX	2.32 d	
FV-C-30	*F.* cf. *inflexum* (FOSC)	HA	2.4 ± 0.61 cZ	2.6 ± 1.40 cZ	3.67 ± 0.95 dZ	0 aX	2.17 c	
FV-C-31	*F.* cf. *inflexum* (FOSC)	MA	1.1 ± 0.61 bY	1.5 bY	1.64 ± 0.05 bcYZ	0 aX	1.06 bc	
FV-C-11	*F. brachygibbosum* (FSAMSC)	HA	2.3 ± 1.16 cZ	2.39 ± 0.81 cYZ	2.6 ± 1.19 cZ	0 aX	1.82 ± 1.15 c	1.36 ± 1.05 c
FV-C-18a	*F. brachygibbosum* (FSAMSC)	WA	0.84 ± 0.10 abXY	2.75 ± 1.39 cdYZ	0 aX	0 aX	0.90 b	
FR-C-29	*F. brachygibbosum* (FSAMSC)	MA	1.55 ± 0.26 bcY	0.35 ± 0.13 aX	0.8 ± 0.01 abXY	0 aX	0.68 ± 1.35 b	0.35 ± 0.65 b
FR-C-24a	*F. brachygibbosum* (FSAMSC)	n/p	0.1 aX	0 aX	0 aX	0 aX	0.03 a	
FV-C-12	*F. clavus* (FIESC)	HA	2.0 ± 0.34 cZ	2.4 ± 1.90 cdZ	2.9 ± 0.15 cdZ	0 aX	1.82 ± 0.45	0.66 ± 0.58
FV-C-13	*F. clavus* (FIESC)	MA	1.0 ± 0.35 bY	1.0 ± 0.35 bY	1.0 ± 0.35 bY	0 aX	0.75	
FV-C-14	*F. clavus* (FIESC)	MA	1.0 ± 0.35 bY	1.0 ± 0.35 bY	1.0 ± 0.35 bY	0 aX	0.75	
FV-C-21	*F. clavus* (FIESC)	WA	1.05 ± 1.21	0.4 ± 0.84 aX	0.8 ± 1.40 bX	0 aX	0.56 ± 0.33	
FV-C-26	*F. clavus* (FIESC)	n/p	0 aX	0.4 ± 0.84 aX	0 aX	0 aX	0.1 ± 0.23	
FV-C-27	*F. clavus* (FIESC)	WA	0.4 ± 1.26 aX	0.65 ± 1.20 aX	1.6 ± 2.07 bcYZ	0 aX	0.66 ± 0.63	
FV-C-32	*F. clavus* (FIESC)	n/p	0 aX	0.14 ± 0.24	0 aX	0 aX	0.04 ± 0.08	

Note: * n/p—non-pathogenic, WA—weakly aggressive; MA—moderately aggressive, HA—highly aggressive. a–d—values with the same lowercase letter are not significantly different among Fusarium isolates; X–Z—values with the same uppercase letter are not significantly different among melon lines with 95% probability according to Duncan’s test.

**Table 4:** Aggressiveness of *Fusarium* isolates against melon seedlings at the stage of the first pair of true leaves (21 dpi).

Strain	Type	Aggressiveness Degree *	Disease Index					
			Variety				Average for Isolate	Average for *Fusarium* Species
			C-IM-7	C-IM-1	C-IM-10	C-IM-6		
	Control		0 aW	0 aW	0 aW	0 aW	0 a	0 a
FV-C-3	*F. annulatum(*FFSC)	n/p	0 aW	0 aW	0 aW	0 aW	0.13 a	0.03 a
FV-C-7	*F. annulatum* (FFSC)	WA	0 aW	0 aW	0 aW	0 aW	0 a	
FV-C-19	*F. annulatum* (FFSC)	n/p	0 aW	0 aW	0 aW	0 aW	0 a	
FV-C-25	*F. annulatum* (FFSC)	n/p	0 aW	0 aW	0 aW	0 aW	0 a	
FR-C-28	*F.* cf. *inflexum* (FOSC)	HA	2.8 ± 0.89 cZ	0.7 ± 0.41 bXY	2.05 ± 0.76 dYZ	0.45 ± 0.28 bX	1.5 d	1.31 c
FR-C-15a	*F.* cf. *inflexum* (FOSC)	HA	2.8 ± 0.89 cZ	0.7 ± 0.41 bXY	2.05 ± 0.76 dYZ	0.45 ± 0.28 bX	1.5 d	
FV-C-30	*F.* cf. *inflexum* (FOSC)	HA	2.8 ± 0.89 cZ	0.7 ± 0.41 bXY	2.05 ± 0.76 dYZ	0.45 ± 0.28 bX	1.5 d	
FV-C-31	*F.* cf. *inflexum* (FOSC)	HA	1.5 ± 1.08 bcXY	0 aW	1.5 ± 1.08 bcXY	0 aW	0.75 bc	
FV-C-11	*F. brachygibbosum* (FSAMSC)	HA	3.0 ± 0.69 cdZ	0.48 ± 0.11 bX	2.7 ± 0.47 eZ	0.23 ± 0.26 aW	1.60 d	1.31 c
FR-C-18a	*F brachygibbosum* (FSAMSC)	MA	1.05 ± 0.34 cX	0 aW	1.05 ± 0.34 dX	0 aW	1.03 c	
FR-C-29	*F. brachygibbosum* (FSAMSC)	n/p	0 aW	0 aW	0 aW	0 aW	0 a	0 a
FR-C-24a	*F. brachygibbosum* (FSAMSC)	n/p	0 aW	0 aW	0 aW	0 aW	0 a	
FV-C-12	*F. clavus* (FIESC)	HA	2.60 ± 0.55 cY	2.0 ± 1.74 cdX	2.0 ± 0.89 dX	0.5 bW	1.78 d	0.505 b
FV-C-13	*F. clavus* (FIESC)	WA	0.73 ± 0.15 bX	0.73 ± 0.15 bX	0.73 ± 0.15 bX	0.11 aW	0.58 b	
FV-C-14	*F. clavus* (FIESC)	WA	0.73 ± 0.15 bX	0.73 ± 0.15 bX	0.73 ± 0.15 bX	0 aW	0.55 b	
FV-C-21	*F. clavus* (FIESC)	WA	0.85 ± 1.05 bY	0.85 ± 1.06 bX	0.85 ± 1.07 bX	0 aW	0.64 b	
FV-C-26	*F. clavus* (FIESC)	n/p	0 aW	0 aW	0 aW	0 aW	0 a	
FV-C-27	*F. clavus* (FIESC)	n/p	0 aW	0 aW	0 aW	0 aW	0 a	
FV-C-32	*F. clavus* (FIESC)	n/p	0 aW	0 aW	0 aW	0 aW	0 a	
Mean			0.99 Y	0.34 X	0.85 XY	0.12 W		

Note: * n/p—non-pathogenic, WA—weakly aggressive; MA—moderately aggressive, HA—highly aggressive. a–e—values with the same lowercase letter are not significantly different among Fusarium isolates; W–Z—values with the same uppercase letter are not significantly different among melon lines with 95% probability according to Duncan’s test.

**Table 5:** Virulence of Fusarium isolates and effects on growth of melon seedlings (21 dpi).

Strain	Type	Aggressiveness Degree	DSI *		EA **, %		*** Proportion of Susceptible Samples, %
			Range	Average	Root	Stem	
Control			0	0 a	0 a	0 a	
FV-C-3	*F. annulatum* (FFSC)	WA	0–0.5	0.21 a	2.8 a	−4.3 a	2.1
FR-C-28	*F.* cf. *inflexum* (FOSC)	HA	0.5–4.0	2.94 d	−38.5 c	−16.7 b	75.8
FV-C-11	*F. brachygibbosum* (FSAMSC)	HA	0.5–4.0	2.44 bc	−16.7 bc	−15.5 b	69.5
FV-C-12	*F. clavus* (FIESC)	HA	0–3.0	1.96 b	−20.4 bc	−16.8 b	20.9

Note: * DSI—disease severity index; ** EA—effect of the action on the root and stem; *** The table shows the average values of the damage index for the sum of all analyzed plants infected with the isolate; a–d—values with the same letter do not have a statistically significant difference with a probability of 95% according to the Duncan test; *—percentage of affected samples with a damage index of 0.5–4.

## Data Availability

The nucleotide sequences have been deposited in the NCBI database (PX512066–PX512084). The original contributions presented in this study are included in the article; further inquiries can be directed to the corresponding author.
